# No Gain No Pain: Relations Between Vestibulo-Ocular Reflexes and Motion Sickness in Mice

**DOI:** 10.3389/fneur.2018.00918

**Published:** 2018-11-12

**Authors:** Erwin Idoux, Michele Tagliabue, Mathieu Beraneck

**Affiliations:** ^1^Center for Neurophysics, Physiology, Pathology, CNRS UMR 8119, Université Paris Descartes, Sorbonne Paris Cité, Paris, France; ^2^Centre National D'Etudes Spatiales, Paris, France

**Keywords:** vestibular, motion sickness, scopolamine, VOR, neurons, mouse, spatial orientation, visuo-vestibular

## Abstract

Motion sickness occurs when the vestibular system is subjected to conflicting sensory information or overstimulation. Despite the lack of knowledge about the actual underlying mechanisms, several drugs, among which scopolamine, are known to prevent or alleviate the symptoms. Here, we aim at better understanding how motion sickness affects the vestibular system, as well as how scopolamine prevents motion sickness at the behavioral and cellular levels. We induced motion sickness in adult mice and tested the vestibulo-ocular responses to specific stimulations of the semi-circular canals and of the otoliths, with or without scopolamine, as well as the effects of scopolamine and muscarine on central vestibular neurons recorded on brainstem slices. We found that both motion sickness and scopolamine decrease the efficacy of the vestibulo-ocular reflexes and propose that this decrease in efficacy might be a protective mechanism to prevent later occurrences of motion sickness. To test this hypothesis, we used a behavioral paradigm based on visuo-vestibular interactions which reduces the efficacy of the vestibulo-ocular reflexes. This paradigm also offers protection against motion sickness, without requiring any drug. At the cellular level, we find that depending on the neuron, scopolamine can have opposite effects on the polarization level and firing frequency, indicating the presence of at least two types of muscarinic receptors in the medial vestibular nucleus. The present results set the basis for future studies of motion sickness counter-measures in the mouse model and offers translational perspectives for improving the treatment of affected patients.

## Introduction

Motion sickness (MS) is a disease that occurs when the brain cannot track the movement of the self in a given environment. Motion sickness is experienced by up to 15% of the humans subjects traveling by air, sea or on ground (1–3). What are the physiological causes for MS? While many theories are still debated (4, 5), it is mostly accepted that MS results from a mismatch between motion-derived neural signals, as for instance a conflict between visual and vestibular inputs experienced while reading in a moving car or on a sailing boat (6). Notably, the conflict between motion-sensitive signals can also be limited to a single sensory modality: vestibular-only motion sickness results from a conflict between semicircular canals signals and otolith signals. Vestibular-only motion sickness incapacitates the brain to integrate angular and linear acceleration in order to efficiently reconstruct the orientation of the head in space (7, 8).

To prevent the onset of motion sickness, medications have been empirically developed and documented for at least a century and probably used for much longer (9). To date, one of the most efficient drugs to prevent in particular space motion sickness (10, 11) is scopolamine (12–14), a muscarinic antagonist commonly administered through transdermal patches. While its molecular effects are well characterized, its putative action on the peripheral and/or central vestibular system, at the neuronal (15) and behavioral levels (14, 16) have still to be specified. Several studies have also tried non-pharmacological approaches to help prevent motion sickness by habituating the system to vestibular stimulation (17–20). Habituation to visual stimulation was also promising because its effects were demonstrated to be long-lasting (1).

While the interactions between the vestibular system, motion sickness and pharmacological treatments have been widely studied in humans, similar studies are conducted on animal models to understand their correlate at the cellular and molecular levels. Here we use the mouse model to investigate the interplay between vestibular reflexes, motion sickness and different counter-measures by addressing several related questions.

1. what are the consequences of motion sickness on the efficacy of the vestibular system?
2. does scopolamine protect mice against MS, as it does in humans?
3. can a non-pharmacological, preemptive adaptation reduce the occurrence of mice MS?
4. what are the direct pharmacological effects of scopolamine on the electrophysiological properties of central vestibular neurons recorded *in vitro*?

We find that motion sickness leads to a general decrease in the efficacy of vestibulo-ocular reflexes (VOR). When administrated before the occurrence of MS, scopolamine decreases the efficacy of the vestibulo-ocular reflexes and prevents the occurrence of symptoms normally associated with MS. Then, we tested the effect of a long-lasting VOR gain-down reduction protocol and validated that this reduction offers a protection against MS. At the cellular level, we demonstrate that muscarinic antagonists have heterogeneous effects on the neuron's electrophysiological parameters suggesting that the action of scopolamine on central vestibular neurons is differentially affecting subpopulations of neurons.

## Materials and methods

### Ethics

Animals were used in accordance with the European Communities Council Directive 2010/63/EU. All efforts were made to minimize suffering and reduce the number of animals included in the study. All procedures were approved by the ethical committee for animal research of the University Paris Descartes (CEEA.34).

### Surgical procedures

Surgical preparation and postoperative care for head implant surgery have been described previously (21, 22). Gas anesthesia was induced using isoflurane. The head was shaved using an electric razor. Lidocaine hydrochloride (2%; 2 mg/Kg) was injected locally before a longitudinal incision of about 2 cm was performed into the skin to expose the skull. A small custom-built head holder (3 × 3 × 5 mm) was fixed using dental cement (C&B Metabond; Parkellinc, Edgewood, NY, United States) to the skull just anterior to the lambda landmark. Following the surgery, animals were isolated and closely surveyed for 48 h. Buprenorphine (0.05 mg/kg) was provided for postoperative analgesia and care was taken to avoid hypothermia and dehydration.

### Behavioral measures

The vestibulo-ocular pathway works as an open-loop: the vestibular signals trigger compensatory eye movements to stabilize gaze in the absence of sensory feedback. As a consequence, any imbalance or modification in the vestibular inputs leads to alteration of the eye movements triggered by head movements. This makes video-oculography the main tool used in hospitals to measure vestibular function. Eye movements were therefore used as a proxy to evaluate the efficacy of the vestibular system by quantification of the vestibulo-ocular reflexes of the mice.

#### Video-oculography procedure

Eye movements were recorded using non-invasive video-oculography (23). The experimental set-up, apparatus and methods of data acquisition are similar to those previously described (22, 24). Briefly, mice were head-fixed at a ~30° nose-down position to align the horizontal canals in the yaw plane (25, 26). Animals were placed in a custom-built Plexiglas tube secured on the superstructure of a vestibular stimulator. The VOR performance was tested in a temperature-controlled room (21°C) with all sources of light turned off except for computer screens. The turntable was further surrounded with a closed box to isolate the animal from remaining light, with a final luminance inside the box <0.02 lux.

To prevent excessive pupil dilatation in dark, a topical application of a combination of pilocarpine (inducing a miosis via local muscarinic stimulation) and Combigan (brimonidine 0.2% + timolol 0.5%, preventing the mydriasis by locally blocking the adrenergic pathways) was used. The addition of Combigan on top of the usually used pilocarpine is necessary to counteract locally the miotic effect of the systemic scopolamine injected in some protocols (cf. Table 1). To avoid introducing a bias between experiments with and without scopolamine, the combination of Combigan and pilocarpine was used in all experiments.

**Table 1:** Experimental protocols.

**Rationale**	**Protocol**	**Injection**		***Provocative rotation***		**VVM**
Control	CTL	Saline		No (*Sham*)		No
Effect of Motion sickness	Rotation	Saline		Yes		No
Effect of Scopolamine	SCO	Scopolamine	Oculomotor testing, pre	No (*Sham*)	Oculomotor testing, post	No
Scopolamine protection against motion sickness	SCO + Rotation	Scopolamine		Yes		No
Behavioral protection against motion sickness	VVM + Rotation	Saline		Yes		Yes

*CTL, Control; SCO, scopolamine; VVM, Visuo-vestibular mismatch protocol*.

#### Vestibulo-ocular reflex tests and analysis

To evaluate the canalar and otolithic contributions to the VOR, different vestibular stimulations were used.

1. The eye movements evoked by an angular stimulation of the horizontal canals (aVOR) were tested. The animal was rotated around a vertical axis with sinusoidal movements at frequency of 0.2, 0.5, 1 Hz with a peak velocity of 25°/s. The angular amplitude of the movement was adjusted accordingly. At least 60 cycles were produced for each frequency. Two parameters were extracted from the recordings: the gain (aVOR_G) and the phase (aVOR_φ). The gain is the ratio between the amplitude of the eye (response) and head (stimulus) rotations. Since the animal is head-fixed to the rotating table, head movements and table movements are identical. The phase is the temporal shift between the eye and table rotations, expressed as ratio of the sinusoidal cycle (2 pi). Details for gain and phase calculation are reported in Carcaud et al. (24).
2. The eye movements evoked by a specific stimulation of the otoliths (maculo-ocular reflexes, MOR) were tested (27) using off-vertical axis rotation (OVAR) as previously described (22). Briefly, the axis of rotation was tilted by 17° with respect to the vertical. Rotations were performed at constant speed (50°/s) for at least 10 rotations both in the clockwise (cw) and the counterclockwise (ccw) directions. Due to the inertial nature of the angular movement detection, a rotation at constant speed elicits a combined canalar and otolithic response at the beginning of the trace, however after a few seconds only the otolithic component remains (22, 28). Since gravitational acceleration acts vertically, this stimulation is equivalent to a continuous rotation (at 0.14 Hz) around the mouse head of a 17° tilted constant linear acceleration stimulus [see Figure 2B in Beraneck et al. (22)]. For horizontal OVAR responses, quick-phases were identified and removed. During rotations, the velocity of horizontal slow phases is modulated (modulation, μ) around a constant bias (β). Both parameters (μ and β) were calculated from the sinusoidal fit of eye horizontal slow-phase velocity using the least-squares optimization of the equation:
  $$\begin{array}{l} \text{S}\text{P}\left(\text{t}\right)=\beta +\mu \cdot \sin\left[2\pi \cdot f_{0}\cdot \left(\text{t}+{\text{t}}_{\text{d}}\right)\right] \end{array}$$

where SP(t) is slow-phase velocity, β is the steady-state bias slow phase velocity, μ is the modulation of eye velocity, f_0_ is the frequency of table rotation, t_d_ is the dynamic lag time (in ms) of the eye movement with respect to the head movement. The bias (Maculo-ocular reflex Bias; MOR_β_) is reported here as the main index of otolithic response (22, 27).

#### Motion sickness generation

**Figure 1:**
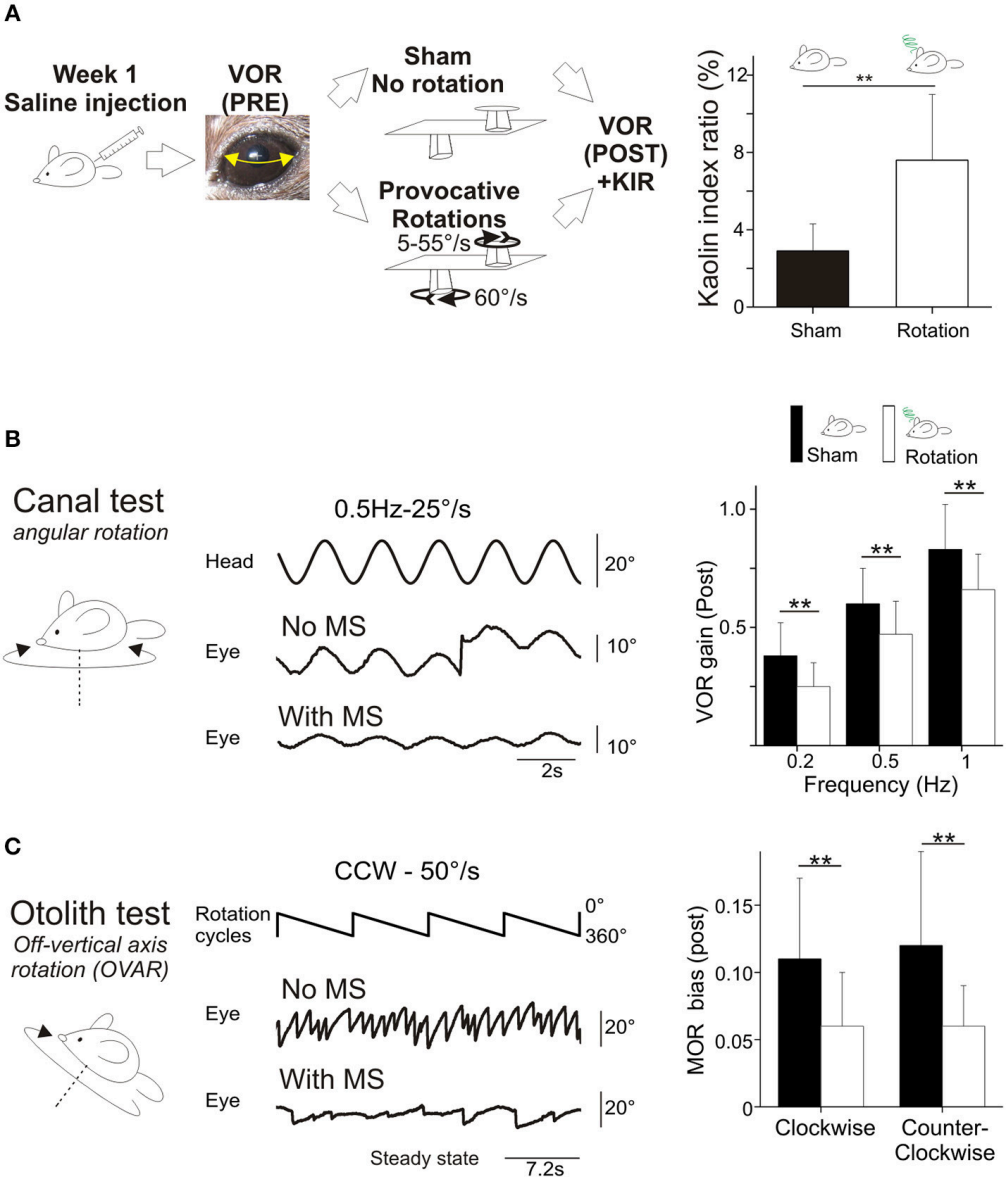
**(A)** Rotation induces motion sickness. Left, scheme of the protocol designed for intra-individual comparison. Animals received saline injection and VOR was tested before and after a *sham* experiment and a provocative double-rotation. Right, *Pica* behavior quantified as a Kaolin Index Ratio was quantified before and after rotation. **(B)** Motion sickness reduces angular horizontal vestibulo-ocular reflex. Left panel, raw traces of the eye movement observed during sinusoidal rotation of the turntable after the *Sham* or provocative rotation session. Right panel, intra-individual comparison of the VOR gain measured with or without MS. **(C)** Motion sickness reduces the maculo-ocular reflex. Off-vertical axis rotation was performed at velocities of 50°/s. A sample of 4 over 10 cycles of 360° rotations at constant velocity are presented. Left, raw traces of the eye movements evoked with or without MS. Right, intra-individual comparison of the MOR gain measured with or without MS. In this and all figures, plots represent mean ± standard deviation. Asterisks indicate statistically significant differences with Holm-Bonferroni correction, *p < 0.05; **p < 0.01; ***p < 0.001 respectively. For table position up is left; for ease of reading, eye position is inverted (up is right).

Motion sickness was induced in mice using a double provocative rotation comparable to the one used in rats by Morita et al. (29). Animals were tested one at a time. Each animal was rotated for 30 min in the home-made motion sickness generating device, under room lighting (300 lux). This device is composed of one central axis rotating clockwise a 30 cm-long arm at 60°/s constant velocity. At the distal extremity of the arm is a second axis, which rotates the box containing the animal counter-clockwise with a sinusoidally-modulated speed (range 5–55°/s; Figure 1A). The box containing the non-restrained mouse had a padded floor. The padding was changed before each test to prevent any olfactory signaling within the box. The top part of the box was transparent.

#### Motion sickness evaluation

Kaolin is a mineral clay commonly used in animal feed. Preparation of a mix of kaolin (Sigma Aldrich **#**18672) and 1% w/w arabic gum (Sigma Aldrich **#**G9752-500G), hereafter referred to as “kaolin”, was similar to that reported by Yu et al. (30). To quantify the occurrence of MS, we measured the changes in alimentary preferences observed following an aversive stimulus. Affected mice eat less of the regular food and instead turn to kaolin, which has no nutritional value.

Each mouse was housed individually for the entire time of the experiment, with *ad libitum* access to water, regular food, and kaolin. Individual consumption of food (F) and kaolin (K) was measured daily. The kaolin intake ratio (KIR) is calculated as K/(K+F) and expressed in percent.

#### Protective protocols

##### Scopolamine dynamics

To test whether the effects of scopolamine were lasting during the entire experiment, we measured the pupil dilation under constant artificial lighting (300 lux) in a separate group of animals (*n* = 12). Animals were injected with scopolamine (Sigma Aldrich **#** S1875-1G; 0.3 μg/g of corporal mass, in saline solution) and the individual duration of the pupil dilation was measured. During this preliminary experiment, the scopolamine effects were found to peak within minutes and then to slowly fade: significant pupil dilatation was seen after 5–7 min and this dilation lasted for at least 90 min, i.e., longer than the duration of the experimental protocols (see Table 1 below). No change in the pupil size was observed when animals were injected with saline in the same configuration.

##### Visuo-vestibular mismatch protocol

To test if decreasing the efficacy of the vestibular system is causally linked to the protective effect of scopolamine, we took advantage of a behavioral protocol recently developed [see Figure 2 in Carcaud et al. (24)] that leads to a decrease of VOR gain. A custom-built device was secured on top of the head holder for 14 days. The device consisted of a “helmet” (size: 2.2 cm width × 1.5 cm depth × 1.5 cm length; weight 2 g) that completely covered the mouse's head. The front of the device was adapted to the mouse anatomy so that the nose was not covered, and its width allowed for grooming and barbering behaviors. To preserve light-dependent physiology and nychthemeral rythm, the device was made of non-opaque plastic with a thickness of 0.3 mm. In addition, 3 mm large vertical black stripes were drawn on the external surface. When the mouse moves its head, the highly contrasted head-fixed stripes generate a visuo-vestibular mismatch (VVM). After 2 weeks, we reported a long-lasting gain-down reduction of the angular VOR of about 50% [range tested 0.2–2 Hz for velocities of 10–50°/s; see results in Carcaud et al. (24)]. Here, we take advantage of this protocol to test the interactions between the VOR and motion sickness.

#### Design of the study

Different procedures were designed to test, on one hand, the functional consequences of motion sickness or of scopolamine on the vestibular system and, on the other hand, the influence of scopolamine or of the visuo-vestibular mismatch on motion sickness.

All behavioral measures reported were performed on *n* = 24 mice. 12 additional animals were used in preliminary experiments to determine the exact parameters used but were not included in the study.

During the first week, the susceptibility of the 24 mice to *provocative rotations* was tested following an injection of a saline solution (Figure 1A). To account for the inter-animal variability and non-specific effects, each animal was tested in 2 sessions: vestibulo-ocular reflexes were tested a first time in the dark (aVOR and MOR testing, *pre*). Then, the animal was put into the motion sickness generating device either activated (i.e., *provocative rotation* condition) or not (*Sham* condition). Finally, the same vestibulo-ocular reflexes were recorded a second time in the dark (aVOR and MOR testing, *post*; Figure 1A).

**Figure 2:**
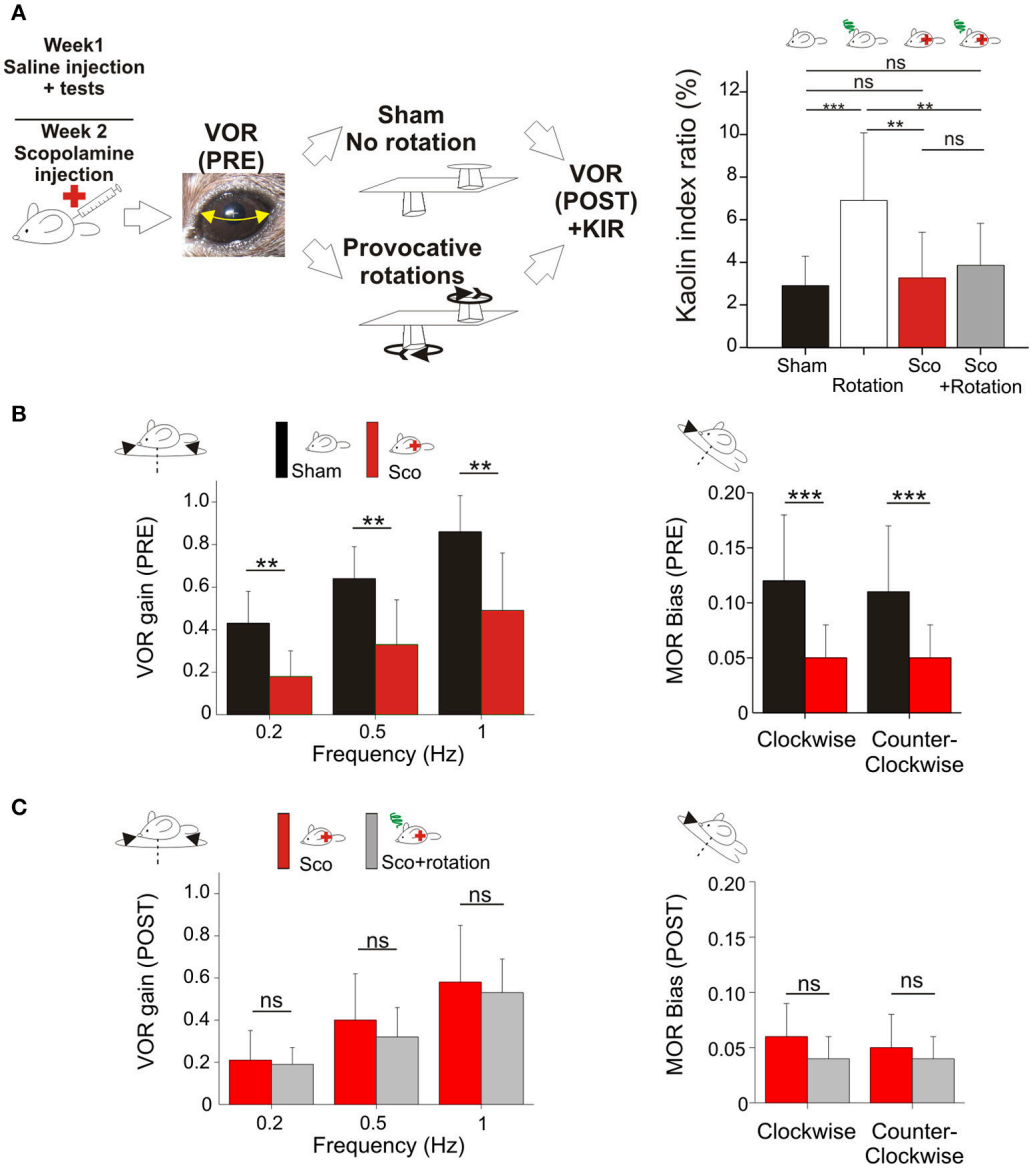
**(A)** Scopolamine protects against motion sickness. Left panels, animals already tested with saline received scopolamine injection; VOR was tested before and after a *Sham* experiment and a provocative double-rotation experiment. Right, *Pica* behavior demonstrated the protective effects of scopolamine against motion sickness. **(B)** Scopolamine reduces vestibular sensitivity. Left, plots of angular VOR gain show significant reduction at all tested frequencies before provocative stimulation (Pre measurements). Right, plots of MOR bias shows significant reduction under scopolamine treatment. **(C)** No additional reduction of vestibular sensitivity was observed following provocative rotation protocol (Post measurements). Left, plots of angular VOR. Right, plots of MOR bias. Asterisks indicate statistically significant differences with Holm-Bonferroni correction, *p < 0.05; **p < 0.01; ***p < 0.001 respectively.

The effects of scopolamine were tested in a subset of mice (*n* = 16). The mice received a scopolamine injection (0.3 μg/g of corporal mass, in saline solution). The mice were then tested again in the *Sham* and *provocative rotations* conditions (Figure 2A).

**Figure 3:**
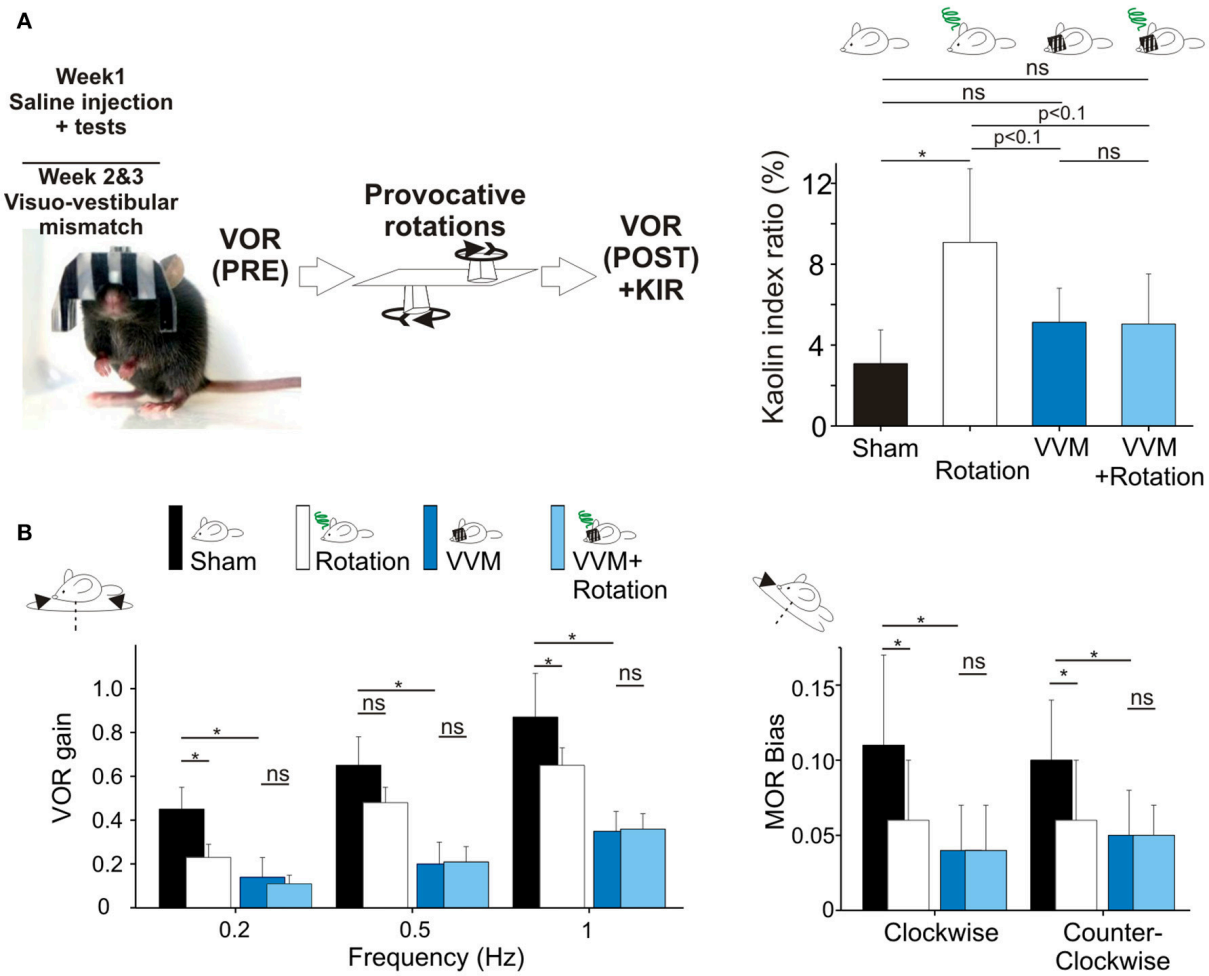
**(A)** Visuo-vestibular mismatch reduces vestibular sensitivity. Left, picture of a mouse during the visuo-vestibular conflict protocol. The helmet is kept for 2 weeks. Right panel, *Pica* behavior demonstrated the protective effect of VVM protocol against MS induced by the double-rotation **(B)**. Left panels, plots of angular VOR of the *n* = 8 mice before the provocative rotations (black and deep blue bars) and after the provocative rotations (white and light blue bars). Right panels, MOR bias ratios in the same conditions. No additional reduction of vestibular sensitivity was induced by the rotation, suggesting protective effects of the VVM protocol. Asterisks indicate statistically significant differences with Holm-Bonferroni correction, *p < 0.05; **p < 0.01; ***p < 0.001 respectively.

To test whether motion sickness could be prevented without scopolamine, the remaining 8 mice were included in the VVM gain-decrease experiment (Figure 3A). After the initial motion sickness tests, the helmet was put on the mouse's head for 2 weeks (see section Visuo-vestibular mismatch protocol). Following this perturbation period, the VOR was recorded immediately after removing the helmet, and again after the *provocative rotation* stimulation. No *sham* condition was recorded to prevent de-adaptation of the VOR.

The different protocols are summarized in Table 1.

### Electrophysiological experiments

To measure the neuronal effects of scopolamine, 220 μm-thick coronal brainstem slices were obtained from 5-week-old male C57BL/6J mice (*n* = 18) (24, 31). A total of 51 medial vestibular nuclei neurons (MVNn) were recorded with patch-clamp electrodes. The artificial cerebrospinal fluid (aCSF) used during the dissection and slicing is composed of (in mM): NaCl (120), NaHCO_3_ (25), NaH_2_PO_4_ (1), KCl (2.5), MgCl_2_ (3), CaCl_2_ (0), glucose (10), sucrose (240). The recording solution differs only for NaCl (120), MgCl_2_ (2), CaCl_2_ (1) and sucrose (0). Analysis of resting discharge parameters, spike shape and classification of type A vs. type B neurons are similar to those previously reported (31). The intrinsic properties, as well as the responses to hyperpolarizing and depolarizing steps were compared between control conditions, or during pharmacological testing by the addition of muscarine (10 μM), or addition of muscarine (10 μM) + scopolamine (10 μM) to the bath. All chemicals were purchased from Sigma-Aldrich.

### Statistics

All mice were first tested during the Control protocol (Figure 1), then during one of the two counter-measure protocols (scopolamine, Figure 2, or visuo-vestibular mismatch, Figure 3). This approach allowed performing statistical analyses based on within-subjects models to account for non-specific and inter-individual variations. Since not all parameters were normally distributed (as tested with a Lilliefors test), we used the same non-parametric paired-test (Wilcoxon signed-rank test) to evaluate statistical significance in all conditions. When appropriate, a one-tail version was used to account for prior knowledge about the alternative hypothesis [e.g., the effect of a treatment on the parameters as the expected reduction of the VOR gain by the VVM protocol, Carcaud et al. (24)]. The thresholds for the statistical tests were adjusted using the Holm-Bonferroni method to account for the numerosity of the planed multiple comparisons. Although adjusted *p*-value thresholds were used to define the level of significance of the statistical tests, for ease of reading we report in the results section the corresponding uncorrected value noted with the ¢ symbol. All results in both the text and the figures are reported as mean ± standard deviation.

## Results

### Effect of rotation on the behavior of control mice

#### Induction and quantification of MS

In response to motion sickness (MS), mice do not vomit (30, 32); however behavioral proxies can be used in rodents to assess the debilitating effects associated with MS. Following the provocative double-rotation protocol, qualitative symptoms such as urination, piloerection or tremor were observed, suggesting that MS had been induced. To quantify the occurrence of MS, we measured the “*Pica”* behavior: changes in alimentary preferences observed following an aversive stimulus (33, 34). Affected mice eat less of the regular food and instead turn to a substance referred to as “*Kaolin”* which has no nutritional value.

Mice food consumption was measured before and after their exposure to the *Sham* condition or *provocative rotation* condition (Figure 1A). The quantity of food and of *Kaolin* was then compared and used to calculate the Kaolin Index Ratio (KIR). As expected, the *Pica* behavior was observed in all mice (*n* = 24) following MS induction and the KIR was significantly increased (Figure 1A; *p* < 0.01^¢^; Table 2a,e for the different protocols).

**Table 2:** KIR for the different protocols.

	**Group 1 vs. Group 2**	**Group 1**	**Group 2**	***p***
		**Mean ±SD**	**Mean ±SD**	
No VVM protocol; *n* = 16 mice	**a** *Sham* vs. Rotation	2.90 ± 1.39	6.90 ± 3.18	0.0014
	**b** *Sham* vs. SCO	2.90 ± 1.39	3.27 ± 2.14	0.2934
	**c** Rotation vs. SCO + Rotation	6.90 ± 3.18	3.86 ± 1.97	0.0026
	**d** *Sham* vs. SCO + Rotation	2.90 ± 1.39	3.86 ± 1.97	0.4627
	**e** *Sham* vs. Rotation	3.08 ± 1.67	9.08 ± 3.64	0.023
VVM protocol; *n* = 8 mice	**f** Rotation vs. VVM + Rotation	9.08 ± 3.64	5.13 ± 1.71	0.062
	**g** *Sham* vs. VVM + Rotation	3.08 ± 1.67	5.13 ± 1.71	0.117

*SCO, scopolamine; VVM, Visuo-vestibular mismatch protocol; SD, standard deviation*.

#### Sustained rotation decreases the efficacy of the vestibular reflexes

To assess the interplay between vestibular responses and MS syndrome, various components of vestibulo-ocular reflexes were tested during passive head-fixed movements performed in the dark.

First, to determine possible non-specific effects of the protocol, mice were tested in a *Sham* condition (put in the device after saline injection, but not rotated; Figure 1A, left panel). There was a diminution of aVOR gain by ~10% (15% at 0.2 Hz, *p* < 0.05^¢^; 9% at 0.5 Hz, *p* < 0.05^¢^; 5% at 1 Hz, *p* > 0.1^¢^) during the second measure. To account for this effect, we compare below and in Figure 1 the different protocols from similar conditions (e.g., *Sham* Pre vs. rotated Pre; *Sham* Post vs. rotated Post).

For all tested frequencies, mice had similar angular VOR gain (aVOR_G; range 0.45 ± 0.13–0.87 ± 0.19; *p* > 0.1^¢^) and phase (range 20.6 ± 7.2 to −2.5 ± 5.8; *p* > 0.1^¢^) responses before the protocols (Pre values). Following the MS protocol however, there was a significant decrease in the aVOR gain (Figure 1B; *p* < 0.01^¢^ for all frequencies). When the responses before and after the rotation were compared, the mean decrease in aVOR gain reached about ~30% at 0.2 Hz and ~20% at 0.5 and 1 Hz (Figure 1B, right panel). There was also significant changes in the timing of the aVOR (aVOR_φ) toward greater phase leads, particularly for the low and middle frequencies (0.2 Hz: Δphase = +5°, *p* < 0.05^¢^; 0.5 Hz: Δphase = +3°, *p* < 0.05^¢^ at 0.5 Hz; 1 Hz: Δphase = +2.5°, *p* > 0.1^¢^ at 1 Hz).

As for the angular VOR, *Sham* condition was first tested for the maculo-ocular reflex (MOR; Figure 1C) and no significant differences were found. Before the MS protocol, the efficacy of the MOR tested in clockwise and counterclockwise direction was similar in all mice (MOR_β_: 0.11 ± 0.06 vs. 0.12 ± 0.07; *n* = 24).

Following the MS protocol however, a significant decrease of ~50% in the efficacy of the MOR was evidenced (*p* < 0.001^¢^; Figure 1C), in both CW (MOR_β_ POST: 0.06 ± 0.04) and CCW directions (0.06 ± 0.03).

Overall, these results demonstrate that the *provocative rotation* induces motion sickness-associated behavior and affects the vestibular system by decreasing its response to motion. This decrease is observed when canal-dependent (aVOR) or otolith-dependent (MOR) reflexes are recorded; suggesting that sensitivity to angular and linear motion is affected.

### Scopolamine prevents motion sickness-related changes in the vestibular system

Scopolamine is known to help preventing motion sickness in humans. To determine if scopolamine has a comparable effect on mice, the effect of an injection of scopolamine on VOR and motion sickness were investigated on a subset of the animals (*n* = 16).

First, the effect of scopolamine on the *Pica* behavior was measured. Injection of scopolamine did not significantly change the baseline of the KIR in *Sham* condition (Table 2b, Figure 2A black vs. red bar; *p* > 0.1^¢^). Then, the protective effects were tested by rotating the scopolamine-injected mice. While the rotation was efficient in provoking motion sickness in the absence of scopolamine (higher KIR with the MS protocol; Table 2a, Figure 2A, white bar; *p* < 0.05^¢^), the KIR remained low when scopolamine was preemptively administrated (Table 2c, Figure 2A, gray bar). The KIR of scopolamine-injected mice after rotation was not different from that of the Control condition (no scopolamine; no rotation; Table 2d, Figure 2A black vs. gray bars; *p* > 0.1^¢^). Thus, while rotation provoked the *Pica* behavior in these mice, preemptive injection of scopolamine protected them against MS.

To determine the interplay between motion sickness and vestibular sensitivity, the VOR and MOR of mice injected with scopolamine was recorded. Following the injection of scopolamine, but in the absence of rotation (no MS induction), the angular VOR gain of mice was decreased (*p* < 0.001^¢^ at all tested frequencies; Figure 2B left panel). The decrease was in range ~60% at 0.2 Hz and ~40% at 0.5 and 1 Hz. Modifications of aVOR also affected the timing of the response with a tendency toward greater phase lead at 0.2 Hz (Δphase = +9°, *p* < 0.10^¢^) and 0.5 Hz (Δphase = +15°, *p* < 0.01^¢^). Notably, this effect of scopolamine was consistently observed in all injected mice tested before provocative rotation. Similarly, the MOR of scopolamine-injected mice was significantly reduced in both CW and CCW direction (Figure 2B, right panel; *p* < 0.01^¢^ for both directions).

Since motion sickness and scopolamine injection both induce reduction of the vestibular gain and increase in phase leads, we asked whether their combination would lead to a greater attenuation of vestibular reflexes. When scopolamine-injected animals were provocatively rotated, the gain of the aVOR was found to stay significantly lower compared to control conditions (*p* < 0.01^¢^ at all frequencies; Figure 2B, left panel). However, there was no additional decrease between the scopolamine and scopolamine+rotation groups (Figure 2C, left panel; all frequencies >0.05^¢^). A similar result was found for the MOR_β_ which was significantly decreased by the scopolamine injection (Figure 2B, right panel, *p* < 0.001^¢^) but was not different in scopolamine-injected mice tested with or without rotation (Figure 2C, right panel). This result demonstrates that the preemptive modification of the vestibular reflexes by scopolamine injection has occluded the effects on the VOR normally observed following rotation (MS induction).

Since the scopolamine injected groups did not show any obvious sign of MS (see behavioral proxies, Figure 2A), we interpret that in saline-injected mice the reduction of the vestibular reflexes could be causally related to the occurrence of motion sickness following rotation. Since scopolamine-injected mice do not suffer from motion sickness, we hypothesize that the diminution of the vestibular sensitivity (by scopolamine injection in this case) could act as a protective mechanism against motion sickness.

### Drugless protection against motion sickness

To test this hypothesis, we took advantage of a new methodology based on a long-lasting (14 days) visuo-vestibular mismatch (VVM, see Methods) which leads to a significant decrease in the gain of the VOR (24). Another subset of the mice (*n* = 8) was initially tested in control conditions and exposed to the provocative protocol. Before VVM, these mice had normal KIR, which again significantly increased following MS induction (Table 2e; *p* < 0.05^¢^). Following these initial tests, the animals were left unperturbed for 48 h, before to start the VVM protocol. This methodology consists in putting on the head of the mouse a device which creates a visuo-vestibular mismatch. For 2 weeks, the animals were left in their home-cage with the apparatus on the head [see Figure 3A and protocol in Carcaud et al. (24)].

How does the VVM protocol affect vestibulo-ocular reflexes? As expected, the VVM protocol significantly reduced the gain of the VOR compared to pre-VVM values at all tested frequencies by >50% (Figure 3B compare black and dark blue bars; *p* < 0.05^¢^). We then compared the maculo-ocular responses of mice before and after the VVM protocol. The MOR responses of the mice post-VVM were also significantly reduced compared to pre-VVM condition by about 50% in both clockwise and counterclockwise directions (*p* < 0.05^¢^ for both directions; Figure 3B right panel). This result demonstrates that the VOR reduction following the long-lasting visuo-vestibular mismatch already reported for the canal-dependent pathway similarly reduces the otolith-dependent pathways, possibly through central mechanisms [see discussion in Carcaud et al. (24)].

Could the reduction of vestibular sensitivity following the VVM protocol prevent motion sickness? As expected the KIR of these mice was increased by the rotations before VVM (Table 2e, Figure 3A, right panel). After the VVM, the KIR was not significantly different from control conditions (*p* > 0.1^¢^; compare black and deep blue bars on Figure 3A). When VVM mice were rotated (light blue bar), their KIR remained low, tended to be smaller compared to that of *Shams* (*p* > 0.1^¢^) and similar to the non-rotated condition (*p* > 0.1^¢^). The KIR in the VVM conditions tended to remain smaller from that of rotated mice (*p* < 0.10^¢^ for both non-rotated and rotated VVM conditions), suggesting a protective effect of the VVM against MS.

To prevent de-adaptation of the reflexes, no *Sham* condition were attempted after removal of the device. VOR and MOR of the VVM mice were thus recorded immediately after removing the device (Pre values), and again immediately after MS rotation (Post values; Figure 3A). The effects of the provocative rotation were evaluated by comparing the PRE and POST effects (Figure 3B dark vs. white bars). As previously described (Figures 1B,C), the provocative rotation induced a reduction of angular VOR and MOR in control condition seen as a significant reduction in most tested conditions (compare black and white bars). Following the VVM protocol, rotation did no longer affect the efficacy of the vestibular reflexes, so that VOR gains and MOR bias all remained low and non significantly different between the VVM and VVM + rotated conditions (Figure 3B, compare blue and light blue bars).

Overall, these results show that rotations trigger in control mice MS symptoms (KIR increase) and lead to a reduction of the aVOR gain with increased phase lead, and to a decrease of MOR bias. Scopolamine or visuo-vestibular mismatch protocols both reduce the efficacy of the reflexes and offer some protection against motion sickness symptoms.

To understand the cellular mechanisms of scopolamine, pharmacological experiments were then conducted on vestibular neurons recorded on brainstem slices.

### Electrophysiological results

#### Scopolamine is specifically acting on MVNn muscarinic receptors

Thirty-two medial vestibular nuclei neurons (MVNn) were recorded in standard ACSF solution. MVNn can be segregated into subpopulations based on the characteristics of the after hyperplorization and inter-spike interval (35, 36). Table 3 summarizes the membrane properties computed from spontaneous discharge (pacemaker activity) of the neurons, i.e., in the absence of any electrical stimulation. Here, from the 32 neurons recorded, 8 were type A neurons characterized by a single, deep afterhyperpolarization (AHP) and 24 were type B neurons characterized by a biphasic AHP. Apart from the *a priori* differences (Concavity, convexity and AHP parameters), only the firing frequency differed significantly (*p* < 0.05) between the 2 subpopulations recorded in control conditions.

**Table 3:** Absence of effects of scopolamine in absence of cholinergic agonists.

	**Type A (*****n*** = **8)**			**Type B (*****n*** = **24)**		
	**Control condition**	**Scopolamine alone (10 μM)**	***p***	**Control condition**	**Scopolamine alone (10 μM)**	***p***
Vm (mV)	−52.76 ± 3.11	−52.69 ± 6.26	1	−51.23 ± 4.69	−50.31 ± 5.70	0.34
F (Hz)	5.61 ± 1.92	1.78 ± 1.24	0.13	13.85 ± 5.89	15.86 ± 11.98	0.57
CV	14.02 ± 8.20	35.86 ± 14.04	0.25	11.86 ± 7.55	24.93 ± 27.94	0.1
AHP (mV)	32.11 ± 7.26	32.40 ± 6.96	0.88	25.83 ± 5.20	25.35 ± 5.61	0.73
Width (ms)	1.70 ± 0.56	1.70 ± 0.52	0.88	0.78 ± 0.23	0.82 ± 0.27	0.12
Concavity (mV)	−2.79 ± 1.55	−6.16 ± 0.75	0.13	−0.05 ± 0.14	−0.62 ± 1.33	0.06
Convexity (mV)	0.31 ± 0.28	0.13 ± 0.06	0.13	0.74 ± 0.56	0.49 ± 0.64	0.38
AHPR (V/s)	0.18 ± 0.11	0.14 ± 0.06	0.38	N/A	N/A	N/A
dAHP (V/s)	N/A	N/A	N/A	6.50 ± 2.41	6.28 ± 3.32	0.85
Resistance Hyperpol. (MΩ)	522 ± 177	514 ± 164	0.38	416 ± 141	378 ± 323	0.08
Resistance Depol. (MΩ)	110 ± 52	153 ± 71	0.13	121 ± 52	120 ± 51	1

*Vm, membrane potential, F, spontaneous discharge frequency, CV, coefficient of variation of the spontaneous discharge, AHP, After HyperPolarization, Width, spike width at threshold, Concavity and convexity: quantification of the shape of the interspike interval, AHPR, quantification of the AHP rectification, dAHP, quantification of the double AHP, Resistance hyperpol: slope of the IV curve in response to hyperpolarizing steps, Resistance depol: slope of the IV curve in response to depolarizing steps. For more details on how these parameters are calculated see (31, 36, 37)*.

Then, scopolamine (10 μM) was applied to the bath. Notably, the addition of scopolamine did not have any effect on either type A or type B neurons (Table 3). Since scopolamine acts as an antagonist of muscarinic receptors, and because muscarinic receptors have been reported in MVNn (38), we hypothesized that this absence of modulatory effect could be due to the *in vitro* slices recording conditions and in particular to the non-activation of the muscarinic receptors. This result suggests that the putative action of scopolamine on MVNn is specific and restricted to its action on muscarinic receptors.

#### Muscarine application can either depolarize or hyperpolarize the cells

**Figure 4:**
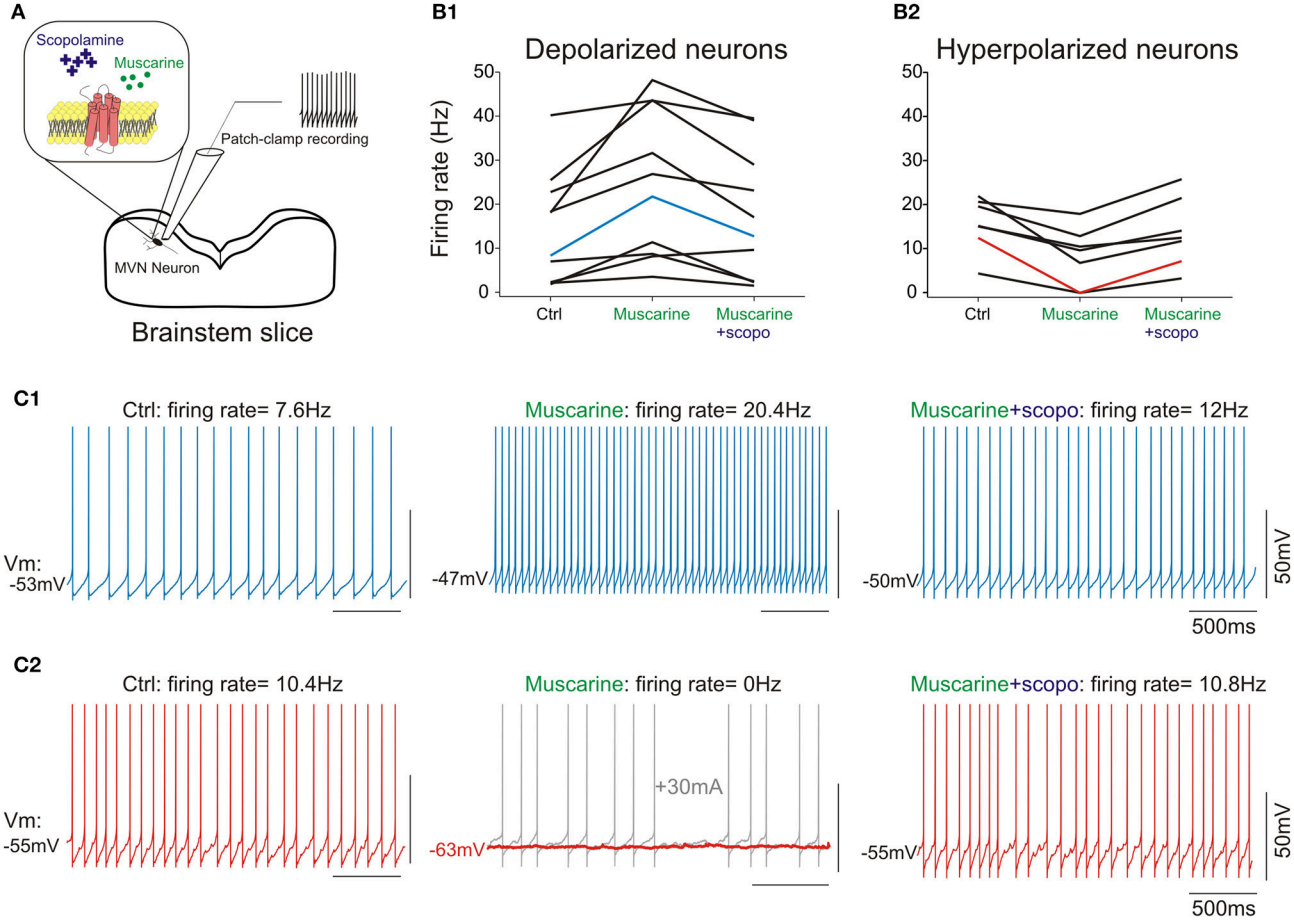
**(A)** scheme of the pharmacology patch-clamp experiment. **(B1)**, cells depolarized by muscarine; **(B2)**, cells hyperpolarized by muscarine. Scopolamine counteracts muscarine effect at cellular level, irrespective of initial response to muscarine. **(C1)** Raw traces from a type B neuron in control condition (left panel), depolarized following addition of muscarine (middle panel), and re-polarized by the addition of scopolamine (right panel). **(C2)** Raw traces from a type B neuron in control condition (left panel), hyperpolarized by muscarine (middle panel) and re-polarized by the addition of scopolamine (right panel). Note that the hyperpolarization induced by muscarine silenced the neuron (middle panel, red trace). A holding current (gray trace) was injected to ensure the neuron was still correctly recorded. Neurons presented in **(C1,C2)** are highlighted in **(B1,B2)**. All numbers and statistics for the electrophysiology experiments are further reported in Tables 3–5.

A second set of 19 neurons (18 type B and 1 type A) was recorded in presence of cholinergic agonists (Figure 4A). Since only one type A was recorded, no interpretation can be made about the effects of muscarine on this subpopulation.

Muscarine depolarized 11 type B neurons by ~3 mV. Application of muscarine strikingly modified the frequency of the spontaneous discharge which nearly doubled (Figures 4B1,C1). In addition, it slightly but significantly increased the amplitude of the AHP and the width of the action potential. Finally, the cellular resistance measured both in presence and in absence of action potentials significantly increased by ~30% (Table 4).

**Table 4:** Effects of scopolamine on neurons depolarized by muscarine.

**Depolarized type B (*n* = 11)**	**I: Control**	**II: Muscarine alone (10 μM)**	**III: Muscarine (10 μM) +Scopolamine (10 μM)**	***p*****-value**		
				**I vs. II**	**II vs. III**	**I vs. III**
Vm (mV)	−48.37 ± 3.97	−45.08 ± 4.72	−47.89 ± 3.68	0.001	0.001	0.52
F (Hz)	13.50 ± 12.54	23.04 ± 16.71	16.48 ± 14.31	0.001	0.003	0.32
CV	24.18 ± 21.91	19.67 ± 33.44	22.31 ± 21.42	0.24	0.102	0.638
AHP (mV)	24.68 ± 4.31	25.89 ± 4.61	26.45 ± 4.87	0.042	0.175	0.0019
Width (ms)	0.81 ± 0.50	0.86 ± 0.51	0.87 ± 0.59	0.0047	0.848	0.186
Concavity (mV)	−0.91 ± 1.87	−0.39 ± 0.76	−1.36 ± 2.10	0.188	0.125	0.813
Convexity (mV)	0.73 ± 0.70	0.90 ± 0.66	0.78 ± 0.53	0.24	0.465	0.7
AHPR (V/s)	0.02 ± 0.03	0.03 ± 0.07	0.03 ± 0.08	0.625	0.625	0.625
dAHP (V/s)	6.36 ± 4.54	5.26 ± 4.13	5.51 ± 4.28	0.0014	0.432	0.105
Resistance Hyperpol. (MΩ)	362 ± 203	470 ± 277	396 ± 232	0.002	0.002	0.375
Resistance Depol. (MΩ)	101 ± 49	128 ± 54	106 ± 49	0.002	0.002	0.492

*Vm, membrane potential, F, spontaneous discharge frequency, CV, coefficient of variation of the spontaneous discharge, AHP, After HyperPolarization, Width, spike width at threshold, Concavity and convexity: quantification of the shape of the interspike interval, AHPR, quantification of the AHP rectification, dAHP, quantification of the double AHP, Resistance hyperpol: slope of the IV curve in response to hyperpolarizing steps, Resistance depol: slope of the IV curve in response to depolarizing steps. For more details on how these parameters are calculated see (31, 36, 37). Statistically significant differences are highlighted in red*.

Conversely, application of muscarine hyperpolarized the 7 remaining type B neurons by ~4 mV (Figures 4B2,C2), while the frequency of the spontaneous discharge was almost halved and the cellular resistance measured in presence and in absence of action potentials decreased significantly by ~40 and 30%, respectively (Table 5).

**Table 5:** Effects of scopolamine on neurons hyperpolarized by muscarine.

**Hyperpolarized type B (*n* = 7)**	**I: Control**	**II: Muscarine alone (10 μM)**	**III: Muscarine (10 μM) Scopolamine (10 μM)**	***p*****-value**		
				**I vs. II**	**II vs. III**	**I vs. III**
Vm (mV)	−53.68 ± 3.17	−57.81 ± 5.58	−54.51 ± 5.90	0.0016	0.0016	0.688
F (Hz)	15.64 ± 6.03	8.25 ± 6.59	13.75 ± 7.80	0.0016	0.0016	0.375
CV	11.65 ± 11.74	9.14 ± 8.25	20.52 ± 15.42	0.938	0.375	0.0016
AHP (mV)	24.90 ± 5.36	23.30 ± 6.66	23.02 ± 7.82	0.375	0.688	left.469
Width (ms)	0.82 ± 0.21	0.87 ± 0.25	0.90 ± 0.27	0.688	0.813	0.234
Concavity (mV)	−0.11 ± 0.29	−0.01 ± 0.03	−0.30 ± 0.80	1	0.5	left.5
Convexity (mV)	1.20 ± 0.86	0.66 ± 0.46	0.69 ± 0.87	0.047	0.688	0.0031
AHPR (V/s)	0.00 ± 0.00	0.00 ± 0.01	0.00 ± 0.00	0.5	0.5	left
dAHP (V/s)	3.95 ± 3.41	3.43 ± 2.97	3.58 ± 3.75	0.469	0.938	0.375
Resistance Hyperpol. (MΩ)	596 ± 300	377 ± 117	462 ± 138	0.0031	0.0031	left.156
Resistance Depol. (MΩ)	173 ± 131	123 ± 102	197 ± 159	0.0031	0.0031	1

*Vm, membrane potential, F, spontaneous discharge frequency, CV, coefficient of variation of the spontaneous discharge, AHP, After HyperPolarization, Width: spike width at threshold, Concavity and convexity: quantification of the shape of the interspike interval, AHPR, quantification of the AHP rectification, dAHP: quantification of the double AHP, Resistance hyperpol: slope of the IV curve in response to hyperpolarizing steps, Resistance depol: slope of the IV curve in response to depolarizing steps. For more details on how these parameters are calculated see (31, 36, 37). Statistically significant differences are highlighted in red*.

#### Scopolamine counteracts an activated cholinergic system

What are the effects of scopolamine on both subpopulations? When applied on the depolarized neurons, scopolamine reversed all the effects of the muscarine application such that neurons membrane potential, frequency of discharge, spike parameters and resistance were all back to normal range and no longer statistically significantly modified compared to control condition (Table 4).

When scopolamine was applied on the hyperpolarized neurons, it also significantly reversed the effects of muscarine on the membrane potential, frequency of discharge, and resistance. Compared to control conditions, only the regularity of the discharge (CV) and interspike interval (Convexity) were still significantly different compared to control condition (Table 5).

Overall, these electrophysiological data show that (i) cholinergic stimulation has opposite effects on specific subpopulations of type B neurons, suggesting that each might express specific type of muscarinic receptors, (ii) scopolamine effects on vestibular neurons depends on cholinergic activation, is direct and specific, (iii) scopolamine acts as an antagonist which completely abolished the various cholinergic responses on all type B neurons tested.

## Discussion

### Rodent models for studying motion sickness using combined genetic, molecular and physiological approaches

Motion sickness is a disease associated with discomfort, and often mistaken with the emetic reflex. While the association of MS and emesis is common in humans, it was demonstrated that rodents actually lack the brainstem neurological components responsible for emesis (32). However, the illness-response behavior known as *Pica* was identified as an analogous to vomiting, observed both in response to intoxication (33) and to provocative vestibular stimuli (29). The *Pica* behavior has since the 90's extensively been used as an index of rat motion sickness [e.g., (34, 39–41)] and was later validated in mice (42, 43). In both species, the causal relation between an intact vestibular system and the *Pica* behavior following challenging rotational stimuli was demonstrated (29, 43). Here, we have shown that in mice, *Pica* behavior can serve as a reliable index of motion sickness induced by a double-rotation paradigm similar to the one originally used in rats (29). We note that other behavioral symptoms such as piloerection, tremble, and abnormal urination were also frequently observed, although not quantified here. The stimulation protocol used is particularly efficient in generating combined canalar and otolithic overstimulation. Because of the possibility to use genetically-engineered mice and to conduct molecular studies, rodent models have recently attracted the attention of many research groups. Wang et al. (44–46) have studied in rats the inter-individual differences and the implication of the vestibulo-thalamic pathway in the habituation to *provocative rotation*. They also demonstrated that otoconia-deficient mice (het) are less susceptible to vestibular MS, indicating the pivotal role of otolithic overstimulation in MS generation. Wang and colleagues (47) took advantage of the mouse model to study the genetic susceptibility to MS by generating MS-susceptible or MS-resistant mouse strains. This recent work suggests the implication of a new protein, the swiprosin-1, in the vestibular-dependent response to MS. Collectively these studies demonstrate that *Pica* behavior constitute a reliable index of MS and reveal the high potential for combined genetic, molecular and physiological approaches in rodent models of MS.

### Relation between vestibulo-ocular reflexes and motion sickness

Many studies have investigated the correlation between VOR characteristics and the susceptibility of the subject to MS, in order to use the VOR as a predictive measurement of MS. Overall, contradictory results were reported regarding angular VOR and occurrence of MS (48, 49). Ventre-dominey and colleagues (50) reported that MS susceptibility co-occurs with decreasing time constant of the VOR and with the increasing eye velocity during otolith-specific stimulation (OVAR); however other studies contradicted this result (51) and rather suggested an implication of the velocity storage in the genesis of MS during OVAR. Recently, Clement and Reschke (52) reported a correlation between MS susceptibility and the phase lead of the VOR at low frequency, with no correlation with VOR gain. Overall, studies in humans suggest a closer relationship between MS and VOR dynamic properties (phase) rather than VOR sensitivity (gain). Notably, human studies were conducted in order to evoke some degree of discomfort, but experiments were stopped just before or as soon as the subject reached sickness (52), limiting the exposure to motion to typically few minutes, which differs from our protocol. We note also that the rotation protocol used here (combination of 2 opposite directions of rotation with sinusoidal variation in angular speed) is more challenging than the protocols used in humans. Our results suggest that a lasting provocative vestibular stimulation leading to the occurrence of MS drives a significant decrease in the gain of vestibulo-ocular reflexes associated with an increase in the phase lead. This decrease in gain similarly concerned the semi-circular canals (angular VOR) and the otoliths (Maculo-ocular reflex), compatible with the hypothesis of a central common mechanism. Reduction of motion sickness following habituation was associated with a decrease in the time constant of the velocity storage (51, 53–56) and there is evidence for angular VOR gain reduction correlated with MS reduction in expert subjects, as for instance in skaters (57) or in sailors (58). A link between a higher aVOR gain and an increase in phase lead was also suggested as an indication of higher seasickness susceptibility (48). Within this framework, the general decrease in gain and increase in phase lead in the vestibulo-ocular responses we report, putatively associated with a decrease in the general sensitivity of the vestibular system, might reduce the sensitivity to the conflicting sensory inputs, and thus putatively help preventing later occurrence of MS.

### Visual and vestibular interactions and motion sickness prevention

If interactions between the VOR main parameters and MS exist, then it might be possible to act on the reflexes to manipulate the susceptibility of the patients. Dai et al. (1) demonstrated in a group of MS-susceptible patients that a visuo-vestibular iterated training protocol could reduce MS sensitivity for several weeks following the habituation sessions. We took advantage of a long-lasting visuo-vestibular mismatch to induce a reduction in the vestibulo-ocular reflexes that again affected equally both canals- and otolith-based reflexes. We demonstrated (24) that this protocol leads to a reduction of the neural responses in the direct VOR pathway. The cellular mechanisms associated to this decrease were a reduction in the synaptic efficiency between the vestibular afferent and the central vestibular neurons and a decrease in the excitability of subpopulations of central vestibular neurons (24). In other term, the long-lasting visual perturbation reduced the brainstem sensitivity to vestibular inputs. Here, we show in mice that the visually-induced reduction in the vestibular reflexes offers a protection against MS. Our results suggest that this effect lasts for at least 3 days, although longer term effects are possible and would deserve dedicated experiments. Overall, our neural and behavioral evidence support the possibility of using visuo-vestibular protocols to habituate susceptible patients to MS induced by vestibular overstimulation or by visuo-vestibular sensory conflicts. For example, since myopic people who wear glasses (but not lenses) have lower angular VOR gains (59), it would be interesting to test whether they are less susceptible to MS than myopic people wearing lenses, and even less than hyperopic people corrected with glasses, whose angular VOR gain is enhanced because of their high positive lenses. Because changes in the efficacy of gaze stabilizing systems are often associated with oscillopsia (60, 61), it would be interesting to study if patients under anti-motion sickness treatments report greater oscillopsia during active head motions.

### Scopolamine effects on the vestibular reflexes and on motion sickness

Scopolamine is well-known as being among the most efficient anti-MS drugs in humans. It is commonly used in particular during space flight as a counter-measure against space motion sickness. In a series of experiments performed on humans in the 80's, Pyykkö et al. (62–64) demonstrated that patches of scopolamine prevented motion sickness by reducing the vestibular and optokinetic gains and suggested that the drug acted on the integrative function of the central vestibular nuclei. More recently, Werts et al. (14) reported a reduction of the angular VOR and caloric response following intranasal administration of scopolamine. Scopolamine had a depressant action on the response of the semicircular canals, postulated to be a combination of peripheral and central effects while it had little effect on the saccular reflex tested with cervico Vestibular Evoked Myogenic Potentials (cVEMP). On the other hand, Tal et al. (65) reported a significant decrease in cVEMP p13 latency following scopolamine administration. Bestaven et al. (16) demonstrated a significant reduction of ~30% of the vestibulo-spinal reflexes following galvanic vestibular stimulation associated with a decrease in balance test and vertical perception. In cat, no direct effect of scopolamine on the VOR was found at low doses, while at high doses the effects were confounded by sedation (66). To our knowledge, our experiments for the first time demonstrate in rodents that the prophylactic effect of scopolamine is associated with a reduction of vestibular sensitivity that concerns not only the semi-circular canal but also the otolith signals. We further show that the preemptive reduction of the vestibular reflexes by scopolamine injection can occlude the reduction of the VOR normally observed following MS. This occlusion suggests that both phenomena rely on a single mechanism or that, if the two processes are distinct, they converge on the same neuronal elements that cannot be adapted below a certain threshold.

### Neuronal mechanisms and motion sickness

What are the neuronal mechanisms associated with MS? Experimental evidence suggests that the processing of divergent sensory inputs in various brain areas (e.g., cerebellum; thalamus) contributes to patients' MS and also impacts the functioning of many cortical areas (67, 68). A key observation which emphasizes the instrumental role of the vestibular system may be, however, that patients with a total loss of labyrinthine function do not get motion sick [review in Lackner (5)]. In addition, in most instances it is the exposure to passive, rather than active, motion that leads to MS (69). In the vestibular nuclei and in the fastigial nuclei of the cerebellum, neurons categorized as “vestibular-only” were demonstrated to differentially encode passive and active movements (70–74). The proposed mechanism is termed “*reafference cancelation*.” It suggests that the vestibulo-cerebellum is using an internal model to predict the consequences of active, voluntary movements and substract this *reafference* signal from the signal sensed by the vestibular organs, termed *exafference* signal. As a result of the substraction of *reafference* and *exafference*, the discharge of vestibular-only neurons would represent the difference between the expected movement and the actual movement. Their discharge thus codes the “unexpected,” passive part of head movements. Vestibular-only neurons are implicated in vestibulo-spinal and vestibulo-sympathic pathway and are nowadays the best candidate for motion sickness generation within the vestibulo-cerebellum (73).

The identification of vestibular-only neurons *in vitro* still remains to be done. However, recent data have suggested that type B neurons constitute the vestibular-projection neurons while type A neurons would constitute the interneurons implicated in local regulation of activity (24, 35). It was also demonstrated that VN neurons that project to the cerebellum and are implicated in vestibulo-cerebellar regulatory loops are glutamatergic, so there is a high probability that vestibular-only neurons and neurons that project on the cerebellum are dominantly type B neurons. Here, all tested type B neurons were found to be modulated by cholinergic stimulation. The presence of nicotinic and muscarinic acetylcholine receptors (mAChR) in the vestibular nucleus with high density in the medial vestibular nucleus is well documented (75–79). Two distinct populations of type B neurons were found based on their modulation by the cholinergic system. Acetylcholine had opposite effects on these subpopulations, suggesting the existence of different receptors. Zhu et al. (38) reported that among the five mAChR subtypes, M2 and M3 may be the most highly expressed in the rat MVN. Interestingly, M2 is linked to the excitatory Gq/11 proteins, while M3 is coupled to the inhibitory Gi/o proteins (80, 81), and both receptors could play distinct roles in regulating vestibular afferent activity onto MVN neurons and activity of cerebellum-projecting neurons (38). The potential of mACHR subtype-specific agonists and antagonists as counter-measures again MS should be the focus of future studies.

## Author contributions

EI and MB: Designed research. EI: Performed research. EI, MT, and MB: Analyzed data and wrote the paper.

### Conflict of interest statement

The authors declare that the research was conducted in the absence of any commercial or financial relationships that could be construed as a potential conflict of interest.
